# Endobronchial Tubercolosis: a peculiar feature of TB often underdiagnosed

**DOI:** 10.1186/2049-6958-7-35

**Published:** 2012-10-22

**Authors:** Lucio Casali, Mariano E Crapa

**Affiliations:** 1 Respiratory Diseases, University of Perugia, Perugia, Italy; 2 Research Fellow, University of Perugia, Perugia, Italy

## 

Endobronchial Tuberculosis (EBTB) is a particular form of TB non easily recognizable, often dangerous for its consequences and potentially a source of spread of infection in the community. Also its definition doesn't find a unanimity. In fact it has been defined as “tuberculous infection of the tracheo bronchial tree with microbial and histopathological evidence” [1] or a” complication of progressive primary Tuberculosis” [2] or, more simply, a “special form of pulmonary Tuberculosis” [3]. It should be stressed that the presence of signs and symptoms qualifies it as a particular expression of a tubercular disease rather than a tubercular infection. Another point of discussion might regard the clinical role of EBTB considering that in the strict sense of a correct classification of TB it might be allocated among the extrapulmonary forms, therefore subjected to diagnostic problems arising from the variability of their clinical manifestations, even though the close continuity with the remaining part of the respiratory system can better draw the attention of the clinicians in comparison with other extrapulmonary localizations. Despite this more favourable position a delay in the diagnosis may be observed with important and seldom severe consequences.

These intrinsic difficulties may have some consequences on the epidemiologic reflexes of EBTB represented by the variability of the data, therefore not always affordable, and by the wide range of percentages reported by different authors. For example Han JK et al. [4] quote a percentage of EBTB between 10 and 40% in patients with active **t**uberculosis that reach 90% in those who exhibit some degrees of bronchial stenosis. On the other hand Chung Hs and Lee JK report an incidence of 5.88% [5], while others authors [6] report an incidence as high as 50% among their patients who were always submitted to bronchoscopy. Analysing the scanty epidemiological references reported by some of these authors it is impossible to distinguish between a real incidence rather than a prevalence derived from a general retrospective investigation and this inaccuracy hampers a correct interpretation of a possible role or trend of EBTB in the general framework of Tuberculosis.

Other details of relevance related to epidemiologic items are the prevalence of origin, the gender and the age of these patients. In fact many sources of literature stress a marked prevalence in young females in Asia or recently migrated from the Far East [3,5,7,10]. Other reports underline other origins from India [11], Turkey [12,13] or from Arabian countries [14].

From a clinical point of view one must keep separated true EBTB from the forms of laryngeal **t**uberculosis which can be originated from the process of expectoration of pooled infected secretions coming from pulmonary cavitations (posterior larynx) or from hematogenous spreading of bacilli [15] to the anterior part of larynx according to the main vascular network. Not always laryngeal TB is easily approached and rapidly diagnosed because the main symptoms draw the attention of the clinicians on the larynx and the signs affecting the voice could be misinterpreted. EBTB, whose signs and symptoms are less evident, can be even more dangerous because of the important and sometimes severe consequences due to its development and complications.

Many entangled and only partly resolved questions regard the pathogenesis and the clinical history of EBTB confounding the chances of a well addressed clinical suspicion. The more indicative symptoms include a barking cough not responsive to the common antitussive drugs, but partly respondent to the steroids [16], bronchorrea when EBTB is part of a cavitary and hypersecretive TB [17], while hemoptysis may occur in a variable amount, seldom massive, and the possible lesion of lymph nodes close to the bronchial branches can cause a thoracic pain of variable intensity [18]. When EBTB is associated to a Right Middle Lobe Syndrome (RMLS) the main symptoms may be represented by: recurrent cough both dry or hawking, purulent sputum, hemoptysis, dyspnea, general weakness, fever and a possible recurrence of pneumonia in the area of the middle lobe (obstructive pneumonia). No symptoms are strictly significant, but a clinical suspicion may arise on their basis. Moreover the level of attention must be high not only because the lesions under the pathological point of view can be much more severe in comparison to a relatively modest clinical feature, but they could rapidly develop towards an acute tracheal obstruction with a possible subsequent respiratory failure [19]. The wide variability due to different situations reflects a possible spectrum of lesions with an evolution in their natural history as pointed out by Hee Soon Chung and Jae Ho Lee [5] who prospectively analyzed the whole series of findings with serial bronchoscopies, starting from the moment of diagnosis just to the completion of treatment. These AA. arrived to the important conclusion that it could be possible to predict the eventual therapeutic outcome during the initial 3 months of treatment for all the subtypes except the tumorous variety which can evolve later into a bronchial stenosis. These AA. previously classified the forms of EBTB into seven categories on the basis of the bronchological observations and subsequent histological findings, which clearly describe the possible variants occurring during the pathological evolutive process. They reported the following forms: actively caseating, edematous-hyperemic, fibro stenotic, tumorous, granular, ulcerative and non specific bronchitic. The AA. concluded recommending a prompt and aggressive treatment in order to prevent further bronchostenotic complications and the tumorous forms require a more strict endoscopic control associated to the same aggressive, early treatment.

It is important to keep in mind the over mentioned considerations because also the differential diagnosis is sometimes complicated. In fact EBTB can simulate other clinical pictures like tumors [11,13], bronchial asthma [9], foreign bodies [7], recurrent pneumonia [20], while in other cases accurate diagnostic procedures are requested when a suspicion of actinomycosis or of bronchial sarcoidosis coexists [21,22].

The diagnostic work-up in case of TB or of a suspected TB is mandatory to try to demonstrate a sputum positivity for *Mycobacterium Tuberculosis*, but the yield of sputum is not as high as in course of a true pulmonary TB with ulcerative lesions and the isolation of mycobacteria despite an accurate sputum examination has been demonstrated in percentage between 16 and 53% [18]. When the suspicion of EBTB is high nuclear amplification tests (PCR) or other methods for amplifying DNA and RNA in a reference laboratory are recommended [18].

The previous remarks on the main characteristics of EBTB stress all the critical points of a non frequent form of TB that can take many clinical and functional complications besides a prolonged risk of spreading the infection within the general population.

In this issue of *Multidisciplinary Respiratory Medicine* is published the work by Ozkaya S. et al. [23]. This work is interesting not only for the clear outline of the many features of this subject treated under the light of the over mentioned classification of Chung HS. and Jae HL. [5], but also for an impressive attempt to draw a relationship between the histopathological findings and microbiology. At first sight the total number of 23 biopsy-proven EBTB patients enrolled from 2003 to 2009 is rather small but this means that, being this work retrospective and based on the registers of their hospital, although reference hospital for TB in Turkey, probably other cases had been lost and the prevalence of EBTB might be higher, but just for this reason a more extensive use of bronchoscopy should be recommended when a case of EBTB is suspected. Another point stressed in this work is the sputum negativity for acid fast bacilli (AFB) in all the samples, while 26% of the patients were positive for AFB in the bronchoalveolar lavage and the highest smear positivity was found in the granular-type. The positivity found in the culture of BAL reached 39.1% of the sample and once more the granular-type cases showed the highest cultural positivity (75%). These results prove the usefulness of this methodology in the work-up related to the diagnostic procedure in the occurrence of EBTB. It has to be remarked that the correct diagnosis was obtained through the histopathologic examinations of the bronchial biopsies. It seems important to recall the importance of the association between the AFB positivity and the granular forms and it is possible to agree with the AA. who hypothesize that the granular type could reflect a phase related with the caseating stage of the granuloma, therefore representing an important therapeutic target. Another relevant item is the AFB negativity in the fibrostenotic stage which probably reflects a late step of this form where the activity of the disease is extinguished and it begins a process of remodelling and scar formation that leads to irreversible modifications responsible for an atelectasis of the dependent areas.

These last considerations again suggest the main steps of the diagnostic procedures which must be based on the detection of all clinical signs and symptoms, on plain radiographs, and on CT scans. After a correct analysis of the images, it becomes worthwhile to carry out a bronchoscopy with BAL and biopsies and microbiologic and histological examinations.

Early diagnosis and a correct therapy may favourably change the course of EBTB. The treatment must be standardized according to the internationally accepted guidelines. The role of steroids is quite controversial, however, and when orally administered they could reduce the inflammatory reactions with positive reflexes both on the early and late bronchial stenosis [23]. Alternatively, a positive role of topical administrations of steroids through repeated bronchoscopic sessions, or with the use of aerosolized steroids and isoniazid [18] have been described. When a medical treatment is not sufficient to prevent the development of a stenosis it is necessary to implant a stent [8] or to turn to surgical treatment with bronchoplastic surgery [24]. Endobronchial laser and curettage can yield interesting positive results provided the correct indications are previously set [25,26].

In conclusion a correct and fast diagnosis at the right time is a milestone that allows the best therapeutic choices able to afford a prevention of future inabilities.
